# Video games as a complementary therapy tool in mental disorders: PlayMancer, a European multicentre study

**DOI:** 10.3109/09638237.2012.664302

**Published:** 2012-05-01

**Authors:** Fernando Fernández-Aranda, Susana Jiménez-Murcia, Juan J. Santamaría, Katarina Gunnard, Antonio Soto, Elias Kalapanidas, Richard G. A. Bults, Costas Davarakis, Todor Ganchev, Roser Granero, Dimitri Konstantas, Theodoros P. Kostoulas, Tony Lam, Mikkel Lucas, Cristina Masuet-Aumatell, Maher H. Moussa, Jeppe Nielsen, Eva Penelo

**Affiliations:** 1 Department of Psychiatry, University Hospital of Bellvitge-IDIBELL, Barcelona, Spain; 2 Ciber Fisisología Obesidad y Nutrición (CIBEROBN), Instituto Salud Carlos III, Barcelona, Spain; 3 Clinical Sciences Department, Medicine School, University of Barcelona, Barcelona, Spain; 4 Systema Technologies, Athens, Greece; 5 MobiHealth B.V., Enschede, The Netherlands; 6 Wire Communications Laboratory, University of Patras, Patras, Greece; 7 Laboratori d'Estadística Aplicada, Departament de Psicobiologia i Metodologia, Universitat Autònoma de Barcelona, Barcelona, Spain; 8 Faculty of Social Sciences and Economics, University of Geneva, Geneva, Switzerland; 9 NetUnion, Lausanne, Switzerland; 10 Serious Game Interactive (SGI), Copenhagen, Denmark

**Keywords:** new technologies, video games, therapy, mental disorders

## Abstract

*Background:* Previous review studies have suggested that computer games can serve as an alternative or additional form of treatment in several areas (schizophrenia, asthma or motor rehabilitation). Although several naturalistic studies have been conducted showing the usefulness of serious video games in the treatment of some abnormal behaviours, there is a lack of serious games specially designed for treating mental disorders.

*Aim:* The purpose of our project was to develop and evaluate a serious video game designed to remediate attitudinal, behavioural and emotional processes of patients with impulse-related disorders.

*Method and results:* The video game was created and developed within the European research project PlayMancer. It aims to prove potential capacity to change underlying attitudinal, behavioural and emotional processes of patients with impulse-related disorders. New interaction modes were provided by newly developed components, such as emotion recognition from speech, face and physiological reactions, while specific impulsive reactions were elicited. The video game uses biofeedback for helping patients to learn relaxation skills, acquire better self-control strategies and develop new emotional regulation strategies. In this article, we present a description of the video game used, rationale, user requirements, usability and preliminary data, in several mental disorders.

## Introduction

Increasingly, more health professionals are becoming interested in innovative and cost-effective treatment approaches. Due to this reason, new technologies for treatment and therapeutic support of various medical illnesses are being commonly developed and applied, from Internet to video games (Barnett et al., 2011; Coyle et al., 2005; Santamaría et al., 2011).

### Video games and health

Video games, a special form of new technologies, were initially developed for entertainment. However, during the last few years, the use of serious games for educational purposes (Barab et al., 2005; Gee, 2003; Squire, 2003) and training (Bergeron, 2008; Rassin et al., 2004) has become more frequent. In the last years, well-produced serious games have been commercialized for improving psycho-education or specific behavioural changes in patients suffering from a variety of medical illnesses, such as: asthma (Bussey-Smith & Rossen, 2007), cancer (Beale et al., 2007), obesity (Barnett et al., 2011), diabetes (DeShazo et al., 2010), stroke (Saposnik et al., 2011) and pain (Hoffman et al., 2000).

### Video games and mental disorders

Given the increasing interest of many national health care systems in extending the accessibility of services and treatment programmes for mental disorders, several new technological strategies have been used, from telemedicine to Internet approaches, vodcast and virtual reality scenarios (Botella et al., 2010; Fernandez-Aranda et al., 2009b; Beintner et al., 2012; Mitchell & Robinson, 2000; Treasure et al., 2010). To date, the use of new technologies has progressively been applied in several mental disorders, including obsessive-compulsive disorders (Mataix-Cols & Marks, 2006), schizophrenia (Bellack et al., 2005), eating disorders (EDs) (Carrard et al., 2011; Sánchez-Ortiz et al., 2011), addictive behaviours (Lee et al., 2007) and anxiety disorders (Difede et al., 2007).

Although several naturalistic studies have been conducted showing the usefulness of serious video games for enhancing some positive attitudes (Beale et al., 2007; Rassin et al., 2004), increasing problem solving strategies (Coyle et al., 2007) and modifying some abnormal behaviours (Walshe et al., 2003), there is a lack of controlled studies in the literature dealing with video games as an additional therapeutic tool for specific mental disorders. Previous pilot studies have suggested that computer games in general could be of help as additional interventions (Wilkinson et al., 2008), in areas such as schizophrenia (Bellack et al., 2005), anxiety disorders (Walshe et al., 2003) and attention deficit hyperac-tivity disorders (ADHD) (Arns et al., 2009). However, those studies have shown limited results and methodological shortcomings (e.g. lack of controlled designs and lack of sample power), making it difficult to expand the obtained results.

### PlayMancer: a video game for treating mental disorders

PlayMancer is an EU initiative to develop a video game prototype for treating specific mental disorders (namely EDs and impulse control disorders). It is being applied at the Department of Psychiatry (University Hospital of Bellvitge, Barcelona, Spain) in mental disorders (mainly EDs and behavioural addictions) and introduces the player to an interactive scenario (named Islands), where the final goal is to increase emotional self-control skills in patients and self-control over their general impulsive behaviours. A multidisciplinary team of clinicians, engineers and programmers have developed this video game, by considering user requirements and emotional reactions as well as personality profiles of the targeted patients.

#### Rationale

Within severe impulse-related disorders, shared dysfunctional emotional regulatory processes and disinhibited personality traits (Alvarez-Moya et al., 2009; Fernandez-Aranda et al., 2006; Harrison et al., 2010; Joos et al., 2011; Krug et al., 2008) are among the most difficult core symptoms to address, even with well-established evidence-based psychological therapies (Fernandez-Aranda et al., 2009a; Jimenez-Murcia et al., 2007; Klump et al., 2004). Due to the lack of effective strategies and adequate psychotherapy tools to remediate these cognitive and emotional processes, we have decided to consider approaches based on serious games. The basic underlying reasons for using such an approach were, on one hand, the potential positive internal characteristics of the video games (namely intensiveness, isolation from outside world, immersive capacity, low resistance to be used) (Griffiths & Meredith, 2009; Wood et al., 2003) and on the other hand, their demonstrated effects in brain activity (Egerton et al., 2009; Han et al., 2011; Koeep et al., 1998).

#### Description of the game and scenario

The scenario which we call “Islands” is based on the idea that the patient is on an island that forms part of several islands in an archipelago. On each island, different activities are available. The activities are linked to varying difficulty levels of game tasks. In this adventure game, the player is confronted with several challenges and situations in order to improve the skills and attitudes that we are trying to change (i.e. problem solving, impulse control, confronting situations associated to frustration and adverse emotion management) (see Table I). As the player completes the various tasks of the game, she/he can advance to higher levels of difficulty. The final objective is not to win, in a classical game manner, but to achieve a greater capacity of self-control. At all times, the patient receives feedback regarding his/her achievements.

**Figure 1:**
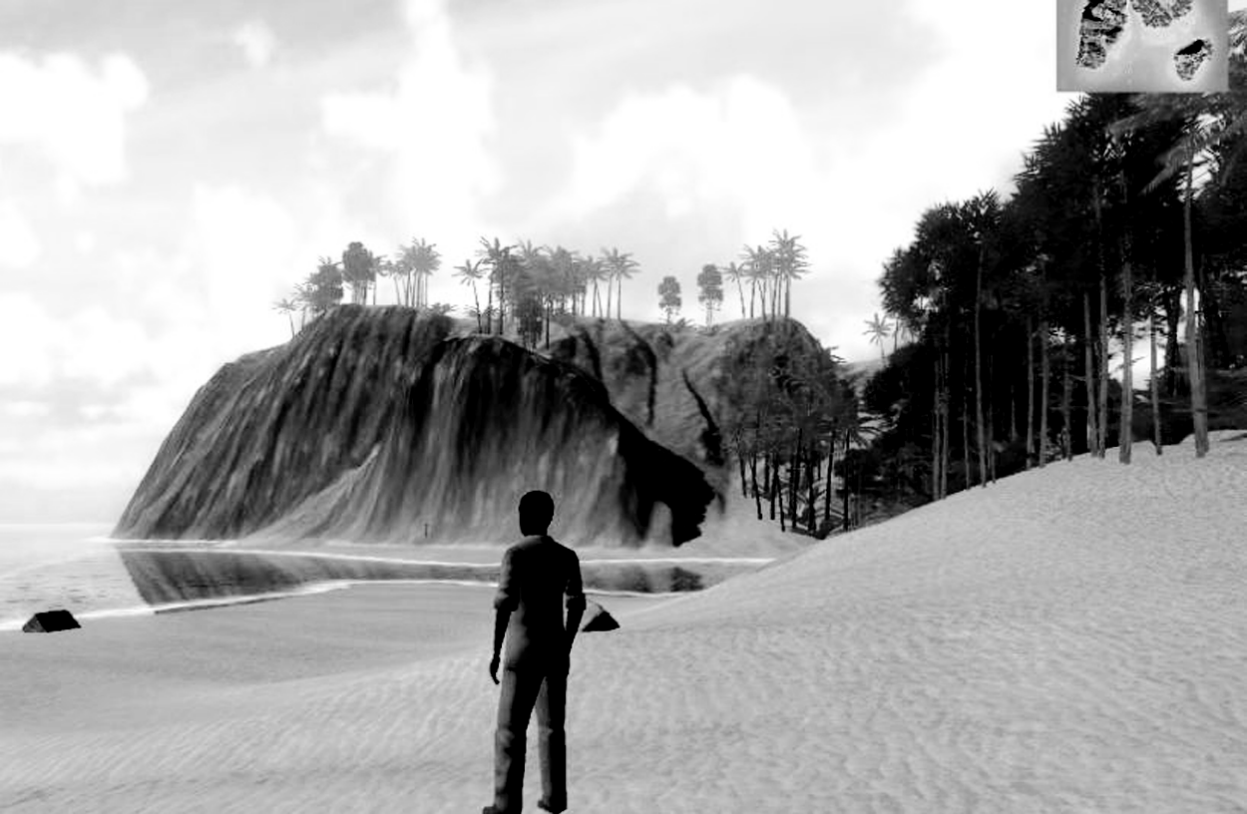
Example of scenario in islands.

The game consists of a series of specifically designed tasks (diving, climbing, learning and training intensive relaxation tasks). Therefore, three prototyped *mini-games* are developed:

- *The face of Cronos:* A game in which the player plans a climbing path up a cliff, while administering his/her resources and avoiding obstacles, produced by emotions.
- *Treasures of the sea:* A game in which the player swims under water, gathering artifacts and balloon fish. The player also has to administer and maintain his/her oxygen level in order to keep on playing. The difficulty of the diving is controlled by the player's current emotions.
- *Sign of the Magupta:* A relaxation game in which a constellation of stars is drawn up corresponding to the level of calmness of the player (Figure 1).

#### Innovative components

Several new components are integrated in this video game platform, such as biosensors (galvanic skin response, oxygen saturation, heart rate (HR) and HR variation, skin temperature, breathing frequency), measured by a stationary measurement system based on the *MobiHealth Mobile*^™^ system, and emotion recognition (anger, joy, boredom) feature extraction algorithms. The bio-signal, facial (facial gestures) and the speech based emotion recognition (Kostoulas et al., 2010a, 2010b) provides detection of the player's emotions while confronted with specific game situations and the triggered emotions. Physiological reactivity and emotional recognition continuously track the emotional state of the player along the video game, while the game automatically responds in return by modifying aspects of the game play difficulty, in a closed loop. When high undesired emotional and/or physiological reactions (e.g. anger feelings, impulsiveness, non-relaxed reactions, frustration, quick and unplanned responses) are detected by the video game, the game immediately directs the avatar to a relaxed area with the goal to calm down. During the whole game session, higher undesired emotional and/or physiological reactions are coupled with greater difficulty to reach the end goals of the video game (e.g. while diving the fishes are more difficult to catch, more obstacles appear in the mini-games). More relaxed and self-controlled reactions are positively reinforced by the game, making the situations easier to handle and the end goals easier to reach.

**Table I:** Game tasks, user requirements and therapy goals in PlayMancer.

Game task:	User requirements	Therapy goals:
The face of Cronos (climbing)	• Lack of stress management • Low tolerance to cope with Increase planning skills adversities • Impulsive behaviours • Strong negative emotional expression in front of minimal stimuli • High physiological reactivity in front of stress • Lack of boredom management	• Increase planning skills • Improve tolerance to cope with adversities • Learning stress management • Learning to delay impulsive responses • Emotional self-regulation • Self-control • Increase physiological and emotional awareness and self-control • Practicing relaxation and breathing techniques • Learning and enhancing coping strategies and planning skills
Treasures of the sea (diving)	• Lack of stress management • Low tolerance to cope with adversities • Impulsive behaviours • Strong negative emotional expression in front of minimal stimuli • High physiological reactivity in front of stress • Lack of boredom management	• Handle to cope with adversities and consequent disappointment • Practice relaxation techniques • Increase planning skills • Learning to delay impulsive responses • Reacting in a more controlled way, from emotional and physiological point of view
Sign of the Magupta (relaxation)	• Strong negative emotional expression in front of minimal stimuli • High physiological reactivity in front of stress • Lack of stress management	• Self-control • Learning and training relaxation techniques • Self-soothing and self-regulation skills (distracting, self-soothing, imagery, relaxation, etc.)

**Figure 2:**
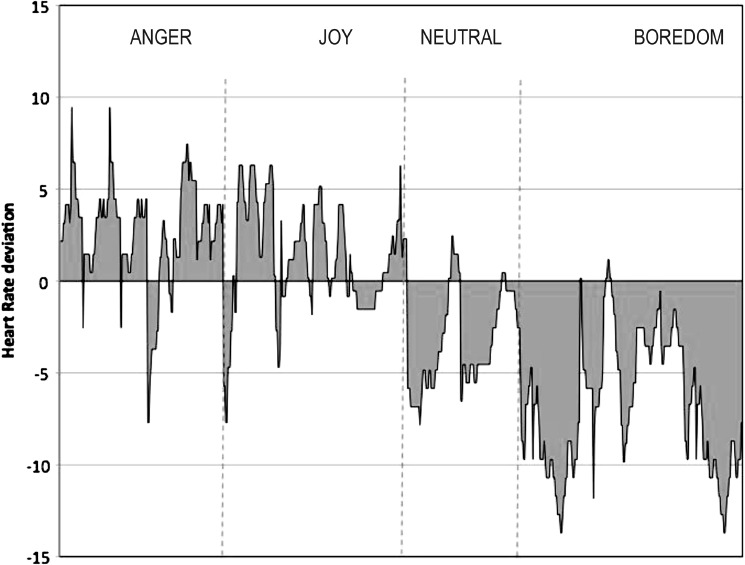
Interaction between emotions (anger, joy, neutral, boredom) and HR correlates. Note: Positive values (above 0) mean over HR rest average score; negative values (below 0) mean under HR rest average score.

Figure 2 shows the average of all the players' interaction between one of the physiological measures (namely HR) and the different emotions recognized by the video game. The deviations from average baseline HR scores are shown. Positive values (above 0) indicate HR values higher than baseline HR, while below 0 indicates values of HR below the baseline. Accordingly, as expected, higher values of excitement (positive HR average deviation scores) are associated with emotions of anger and joy, whereas low values of excitement (negative HR average deviation scores) mainly are associated with boredom and a neutral condition.

#### Design and procedure

In mental disorders (in this case EDs and impulse control disorders), controlled prospective longitudinal studies have been conducted since September 2010, at the Department of Psychiatry (University Hospital of Bellvitge, Barcelona, and CIBEROBN Research Unit). The Islands video game is used as an additional therapeutic tool (combined with standard psychological approaches), with consecutively admitted outpatients. To avoid video game side effects (Anderson et al., 2008; Dworak et al., 2007; Kim et al., 2008), the whole process is controlled and supervised at all times (the game is applied in a coach mode monitored in our hospital, under supervision by a therapist).

Each session consists of exposure to the above-mentioned video game, where the performance of patients is collected for 20 min. Before and after each video game, 3-min of relaxing music is provided to the experimental subjects. Each session is carried out once a week, coinciding with the day that the patients come to the traditional therapy. According to the length of the traditional therapy, 12–14 weekly sessions of video game are applied.

**Figure 3:**
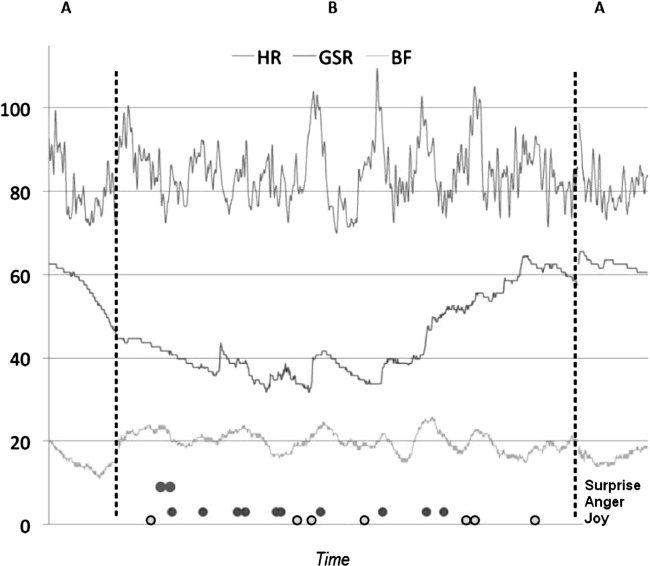
Physiological and emotional reactivity of a single patient, during one session of video game (A–B–A design). Note: GSR, galvanic skin response; BF, breathing frequency; design: A: baseline 3 min; B: gaming 20 min; A: baseline 3 min).

Figure 3 shows the physiological and emotional reactivity of a single patient, during a session. The physiological reactions show activation during the play time and certain emotions are recognized. X axis shows the time of playing, and the Y axis shows amplitude and type of emotions.

#### Inclusion–exclusion criteria

The inclusion criteria used for the whole project study were a diagnosis of bulimia nervosa, binge ED or pathological gambling, according to DSM-IV criteria (APA, 2000), and an age between 18 and 45 years. The exclusion criteria were the following: primary psychiatric or neurological disorders that can interfere with the game performance (e.g. psychotic disorders, bipolar disorders, major depressive disorders, substance abuse-dependence disorders, etc.); active pharmacological therapy that may interfere with the game performance; current or lifetime diagnosis of behavioural technological addictions and current or lifetime comorbid diagnostic of ADHD.

#### Usability of PlayMancer

To analyse the usability and comfort when using the Video game and the connected multiple biosensors and recording camera-microphone, the system usability scale (SUS; Brooke, 1996) was completed by the individuals. SUS is a simple, 10-item Likert scale giving a global view of subjective assessments of usability. SUS scores have a range of 0–100.

**Figure 4:**
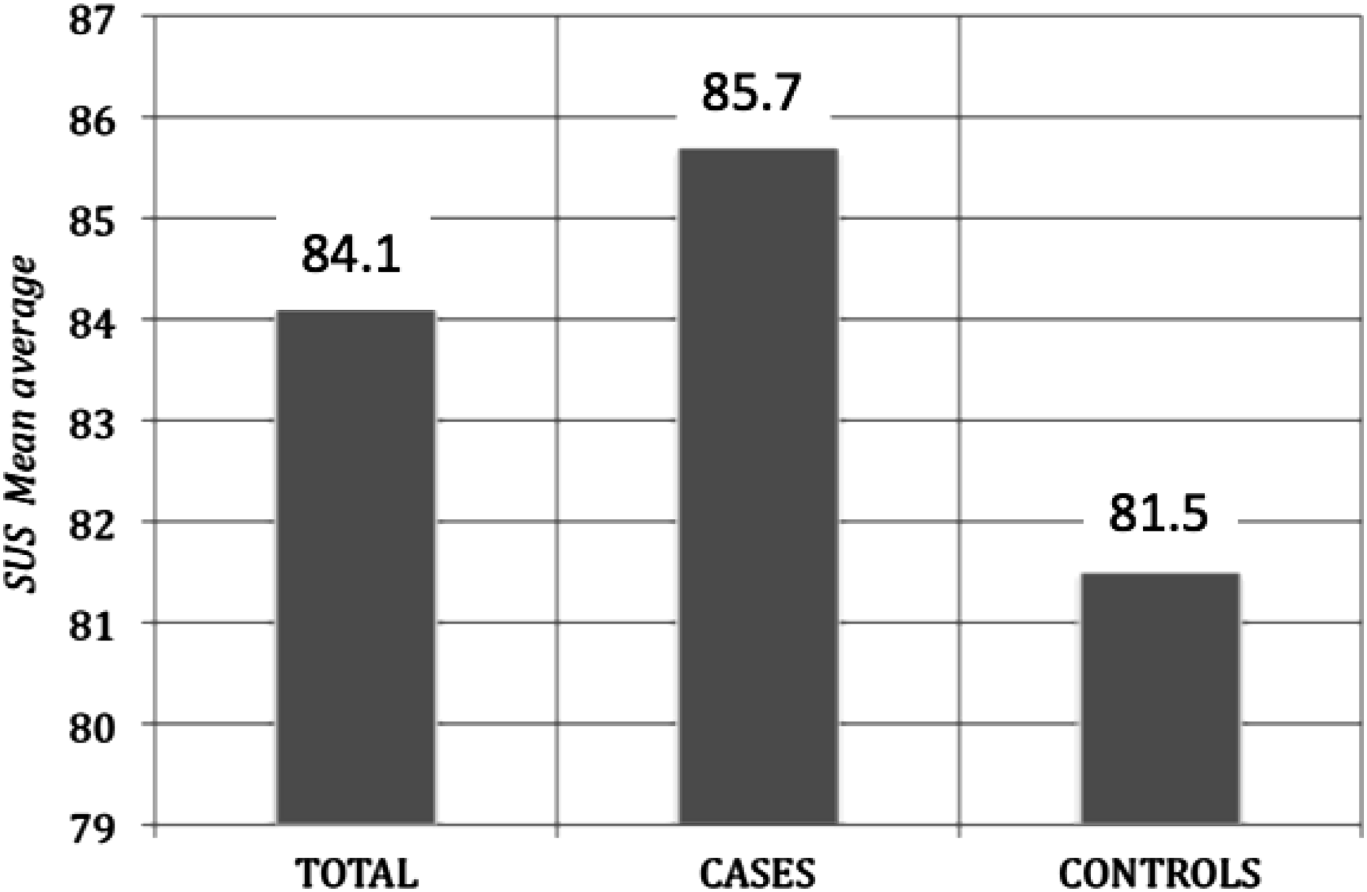
Average usability scores (by means of SUS) assessed in 38 individuals (24 mental disorders and 14 healthy subjects).

Between May and June 2010, we conducted a pilot test study with 24 patients (12 EDs and 12 pathological gamblers (PGs)) and 14 healthy controls-comparison group who volunteered to participate in the study, and who signed an informed consent to participate. All of them were screened for video game and Internet addiction, but also for general health state. The gender proportion for the whole sample was 51.8% (males) *vs.* 48.6% (females), and the mean age was 32.3 years old (SD 9.3), ranging from 18 to 49; 46.3% of the individuals were non-married and 65.5% had secondary or university studies. As shown in Figure 4, we found a mean of the average score estimation of 84.1% (SD 13.2) in the average usability scores (as measured by the SUS) after using Islands. No significant differences were found between patients and controls (Mann–Whitney U = 117.5; *p* = 0.922) on average SUS scores.

## Discussion

Although new technological approaches have increasingly been used as therapy for mental disorders, there is a lack in the literature regarding use of video games and controlled studies analysing its effectiveness. The few studies attempting to analyse video games, as combined therapy in mental disorders, are simply descriptions of series of cases or naturalistic studies with important methodological limitations. However, the internal features of this type of technologies may encourage clinicians and technicians to continue investigating this field.

Taking into account our clinical experience using and developing video games, the most important lessons that we have learned are the following: (a) the acceptance of mental disorder patients is very high for participating and using video games; (b) this approach is a good platform to work out underlying attitudinal and emotional problems, that otherwise are difficult to treat in mental disorders (such as impulsiveness, emotional regulation, frustration), in an intensive and motivating way; (c) internal characteristics of video games make it feasible to apply techniques that tend to be difficult to apply in those patients, such as controlled intensive exposure, immediate positive and negative reinforcing, complex biofeedback approach, and real time monitoring of physiological-emotional reactions.

**Figure 5:**
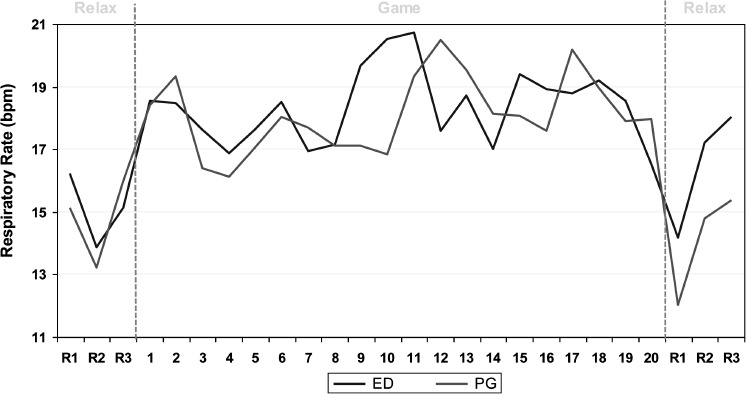
Respiration frequency of five EDs and five PGs, along one session of video game (A–B–A design). Note: bpm, breathing per minute; design: relax (A): baseline 3 min; B: game (B): 20 min; relax (A): baseline 3 min.

Currently the PlayMancer evaluation trials are still ongoing, but initial results, based on more than 40 mental disorder outpatients, seem to indicate that: (a) ED and impulse control disorder patients feel comfortable using such a video game (usability over 85%); (b) a source of stress (such as specific parts of Islands) is able to trigger high physiological and emotional reactions in mental disorder patients, and is moreover over expressed when compared with healthy controls; (c) negative and positive emotions (namely anger and joy, respectively) are positively linked with higher physiological reactivity in mental disorders; (d) as shown in other studies (Pawlow et al., 2003), relaxation and intensive biofeedback may significantly reduce the tension triggered by the game, this is illustrated in Figure 5, which presents the respiration frequency of five ED patients and five PGs during one session of the video game. The respiration rate increases during the game play and decreases during the relaxation time (before and after).

Looking at the short-term effects, after using this video game strategy, the patients started to show new coping styles with negative emotions in normal stress life situations, additional generalization patterns and more self-control strategies when confronted with them. As shown in previous studies (Freidenberg et al., 2002; Goldin & Gross, 2010; Kozasa et al., 2012), working with underlying attitudinal and emotional factors, in EDs and impulse control disorders, we may reduce their potential maintaining capacity and therefore enhance the long-term effectiveness of traditional therapies. In that case, new technological approaches, namely video games, can certainly be one positive option.
